# Estrogen receptor 1 gene polymorphisms are associated with metabolic syndrome in postmenopausal women in China

**DOI:** 10.1186/s12902-018-0289-4

**Published:** 2018-09-14

**Authors:** Lingxia Zhao, Xuemei Fan, Lin Zuo, Qiang Guo, Xiaole Su, Guangxia Xi, Ziyan Zhang, Jianlin Zhang, Guoping Zheng

**Affiliations:** 1 Department of Biochemistry and Molecular Biology, Shanxi Medical University, Taiyuan, No.56, Xinjian South Road, Taiyuan, Shanxi 030001 People’s Republic of China; 2 Department of Endocrinology Medicine, Dayi Hospital Affiliated to Shanxi Medical University, Taiyuan, Shanxi 030032 People’s Republic of China; 3 Department of Physiology, Shanxi Medical University, Taiyuan, Shanxi 030001 People’s Republic of China; 4 Department of Medical Statistics, School of Public Health of Shanxi Medical University, Taiyuan, Shanxi 030001 People’s Republic of China; 5 Renal Division, Shanxi Medical University Second Hospital, Shanxi Kidney Disease Institute, Taiyuan, Shanxi 030001 People’s Republic of China; 6 Centre for Transplantation and Renal Research, University of Sydney at Westmead Millennium Institute, Sydney, NSW 2145 Australia

**Keywords:** Metabolic syndrome, Postmenopausal women, Estrogen receptors 1 and 2, Gene polymorphisms

## Abstract

**Background:**

Metabolic syndrome (MetS) includes obesity, diabetes, dyslipidemia and hypertension. Its incidence is rapidly increasing worldwide, particularly in postmenopausal women. Estrogens regulate glucose homeostasis and lipid metabolism via estrogen receptors 1 (ESR1) and 2 (ESR2). The current study aimed to elucidate associations of MetS with ESR1 and ESR2 gene polymorphisms in postmenopausal Chinese women.

**Methods:**

This case-control study included 304 postmenopausal women (154 and 150 control and MetS patients, respectively). Clinical indicators related to MetS were assessed. Two ESR1 (PvuII and XbaI) and two ESR2 (RsaI and AluI) polymorphisms were evaluated by polymerase chain reaction (PCR)-restriction fragment length polymorphism analysis.

**Results:**

ESR1 polymorphisms were significantly different between MetS patients and healthy controls. G allele frequency for the XbaI polymorphism was significantly higher in patients than in control patients (*p* = 0.004, OR = 1.610, 95%CI 1.169–2.18). The haplotypes A–T (*p* = 0.015) and G–C (*p* = 0.024) showed significant differences. The minor alleles of the XbaI and PvuII gene polymorphisms in both homozygous and heterozygous forms showed associations with elevated waist circumference, fasting serum insulin and HOMA-IR. The minor G allele in homozygous and heterozygous forms of the RsaI and AluI gene polymorphisms showed associations with elevated total cholesterol and LDL-C.

**Conclusions:**

In postmenopausal Chinese women, ESR1 polymorphism and the haplotypes A–T and G–C of XbaI–PvuII are associated with MetS, unlike ESR2 polymorphisms. Patients harboring the G allele of XbaI have elevated BMI, waist circumference, systolic and diastolic BP, FBG, HOMA-IR, total cholesterol, TG, LDL-C and NAFLD (%), and reduced HDL-C.

**Electronic supplementary material:**

The online version of this article (10.1186/s12902-018-0289-4) contains supplementary material, which is available to authorized users.

## Background

Metabolic syndrome (MetS) constitutes a threat to human health, with estimated prevalence rates of 17–40 and 16.3% in the Middle East and North Africa, respectively [1]. Its prevalence has also increased from 23.8% between 2000 and 2005, to 27% between 2010 and 2015 in China [2]. MetS increases the risk of coronary artery disease, stroke, fatty liver disease, diabetes mellitus (DM), insomnia, and several cancers [3, 4]. Adult Treatment Panel III (ATP III) proposed that MetS encompasses at least three of the following ailments: abdominal obesity, high triglyceride (TG) levels, low high-density lipoprotein cholesterol (HDL-C) amounts, high blood pressure (BP), and high fasting blood glucose (FBG) [5]. All MetS variations increase the risk of cardiovascular disease (CVD). CVD incidence and mortality are significantly reduced in premenopausal women compared with men, but this difference gradually diminishes after menopause [6], with a corresponding increase in MetS incidence [7].

Hormones have critical functions in MetS pathogenesis and progression. Estrogen is associated with many aspects of MetS [8] such as glucose tolerance, fat metabolism, and BP [9]. Estrogen exerts its physiological functions in combination with estrogen receptors (ERs). ERs are nuclear hormone receptors (transcription factors) found in macrophages, adipose cells, vascular smooth muscle cells, and vascular endothelial cells [10, 11]. Estrogen receptor-α (ESR1) and β (ESR2) represent two known estrogen receptors that have been assessed using transgenic ERa (ERa^_^/^_^) and/or ERb (ERb^_^/^_^) knockout mice [12, 13].

The subtypes of ER, ESR1, and ESR2 are distinct in structure and function (likely in carbohydrate and lipid metabolism) [14]. ESR1 and ESR2 are found on chromosomes 6q25.1 and 14q23.2, respectively [15]. Single nucleotide polymorphisms (SNPs) in ESR1, such as PvuII (rs2234693) and XbaI (rs9340799), are involved in MetS [16]. As for ESR2, the risk of MetS is determined by the RsaI (1082A > G) (rs1256049) and AluI (1730A > G) (rs4986938) polymorphisms [17]. ESR1 and ESR2 SNPs are associated with metabolic factors in Western populations [18]. Here we assessed associations of four representative ESR1 and ESR2 polymorphisms with MetS and related phenotypes in postmenopausal Han Chinese women.

## Methods

### Study population and laboratory measurements

This case control study was performed between January and November 2016 at the medical center of Shanxi Dayi Hospital (Taiyuan, China). Three hundred and four postmenopausal women aged 46–71 years were enrolled. Inclusion criteria were: ≥60 years old; permanent menstrual period discontinuation because of natural menopause.

At baseline, the subjects were assigned to 2 groups, including the control (154 healthy individuals; mean age of 63.0 ± 4.1 years) and MetS (150 MetS patients; mean age of 62.9 ± 4.8 years) groups. All subjects were Han Chinese and of the same ethnic group. All cases were diagnosed according to the Chinese type 2 diabetes Prevention Guide (2013) diagnostic guidelines [19]. MetS was reflected by ≥3 of the following parameters: waist circumference ≥ 85 cm; FBG ≥6.1 mM or 2 h post glucose–load blood glucose ≥7.8 mM or known diabetes; serum TG ≥1.70 mM; HDL-C < 1.04 mM; and BP ≥130/85 mmHg or treated hypertension. Non-alcoholic fatty liver disease (NAFLD) diagnosis was performed by abdominal ultrasonography, as described previously [20]. Exclusion criteria were: severe psychological ailments, physical disability, any type of malignancy, or history of smoking, heart failure, myocardial infarction (MI), type I diabetes, kidney failure, and chronic liver disease. The subjects were not undergoing hormone replacement.

### Data collection

The main measurements and clinical data of the participants, including age, height, weight, body mass index (BMI), waist circumference, and systolic and diastolic BPs, were measured and recorded by specifically trained individuals. Waist circumference measurement was carried out in the horizontal plane at the midpoint between the lower border of ribs and the iliac crest. Systolic and diastolic BPs were obtained in right upper arm twice following a 15 min-rest with the patient sitting. Measurements were performed at 5 min intervals and averaged.

FBG, hemoglobin A1C (HbA1C), TG, total cholesterol (TC), HDL-C, low density lipoprotein (LDL-C; direct measurement), estradiol (E2), and fasting serum insulin levels in all participants were measured from fasting blood samples on an AutoAnalyzer (AU5400, Olympus, Tokyo, Japan), with reagents and calibrators provided by the manufacturer. Abdominal ultrasonography was conducted by specialized radiologists based on unified NAFLD diagnostic criteria. Insulin resistance (homeostasis model assessment of insulin resistance, HOMA-IR) was determined for each subject.

This study followed the Declaration of Helsinki, as well as institutional regulations regarding human subjects. Written informed consent was provided by all participants. The study protocol had approval from the ethics committee of the Shanxi Da Yi Hospital Institute for Biological Sciences, Chinese Academy of Sciences.

### Genomic DNA extraction and genotyping

Venous blood (2 ml) specimens were obtained in EDTA anticoagulation tubes and used for DNA extraction. Genomic DNA extraction from 200 μL whole blood was carried out with the TIANamp Blood DNA Kit (Tiangen Biotech, China), as instructed by the manufacturer.

The PvuII (c454-397 T > C) and XbaI (c454-351A > G) polymorphisms in ESR1 as well as the RsaI (1082A > G) and AluI (1730A > G) polymorphisms in ESR2 were assessed by polymerase chain reaction- restriction fragment length polymorphism (PCR-RFLP). A 1374 base pair (bp) fragment was amplified for the PvuII (c454-397 T > C) and XbaI (c454-351A > G) polymorphisms using the following primer pair: upstream, 5′-CTGCCACCCTATCTGTATCTTTTCCTATTCTCC-3′; downstream, 5′-TCTTTCTCTGCCACCCTGGCGTCGATTATCTGA-3′. A 476-bp fragment for the RsaI (1082A > G) polymorphism was amplified using the following primer pair: upstream, 5′-TTCTGAGCCGAGGTCGTAGT-3′; downstream, 5′-CCAGGACTTTGTTCCCACTC-3′. A 391-bp fragment for the AluI (1730A > G) polymorphism was amplified with the following primer pair: upstream, 5′-GCACCTTTTTGTCCCCATAGTAAC-3′; downstream, 5′-CTATGGCTTCCTCACACCGAC-3′. PCR was carried out on an ABI Prism 7500 sequence system (Applied Biosystems, USA) in 25 μl reactions, containing 5 μl genomic DNA, 10 μl of Taq PCR Master mix (2×), 2 μl of each primer, and 8 μl PCR buffer (Tiangen Biotech, China), as follows: 94 °C (4 min); 35 cycles of 94 °C (30 s), 57 °C (40 s) and 72 °C (40 s); 72 °C (7 min). Digestion of PCR products was performed overnight using XbaI (expected fragments of 1374, 393, and 981 bp), PvuII (expected fragments of 1374, 438, and 936 bp), RsaI (expected fragments of 476, 183, and 293 bp), and AluI (expected fragments of 391, 144, and 247 bp) (Biolabs, New England) at 37 °C. All the digested fragments were electrophoresed on 2% agarose gels containing Goldview. The genotypes of 10% specimens were verified by direct sequencing.

### Statistical analysis

SPSS 20.0 was employed for statistical analyses. Normally distributed continuous data are mean ± standard deviation (SD). Genotype differences were assessed in MetS patients by analysis of variance. Baseline features between the MetS and control groups were evaluated by Student *t*-test. Comparisons among multiple groups were performed by ANOVA with Bonferroni correction. Hardy–Weinberg equilibrium test, allele genotype frequency assessment, and haplotype evaluation were carried out online with the SHEsis platform. Genotype frequency, allele frequency, and haplotype were analyzed by the chi-square test and odds ratio (OR) assessment. Multivariable logistic regression was performed to adjust ORs for possible confounders. *p* < 0.05 indicated statistical significance. The function pwr.chisq.test() in the R package was used to calculate statistical power.

## Results

### Demographic and clinical variables

The MetS and control groups included postmenopausal women, with similar ages in both groups. All clinical parameters in Table 1 showed statistically significant differences between the MetS and control groups. Elevated BMI values and waist circumferences were observed in MetS patients, who also showed higher mean systolic and diastolic BPs compared with controls. In addition, the MetS group had markedly higher FBG, hemoglobin A1c, fasting serum insulin and HOMA-IR levels, and worse lipid profile. Furthermore, prevalence of nonalcoholic fatty liver disease was far higher in MetS patients compared with controls.

**Table 1:** Patient clinico-biochemical features

Variables	Metabolic syndrome (*n* = 150)	No metabolic syndrome (*n* = 154)	P
Age (years)	63.0 ± 4.1	62.9 ± 4.8	0.751
BMI (kg/m^2^)	25.4 ± 2.9	22.5 ± 3.0	<0.001
Waist circumference (cm)	88.3 ± 5.2	81.9 ± 2.5	0.001
FBG (mM)	7.1 ± 1.4	5.2 ± 0.7	<0.001
HbA1C (%)	6.7 ± 0.9	5.2 ± 0.4	0.003
Systolic blood pressure (mmHg)	148.1 ± 6.9	122.1 ± 4.9	<0.001
Diastolic blood pressure (mmHg)	92.3 ± 4.5	71.8 ± 5.7	0.002
Triglycerides (mM)	2.4 ± 1.1	1.8 ± 1.2	<0.001
Total cholesterol (mM)	5.1 ± 1.0	4.6 ± 1.1	<0.001
HDL-C (mM)	1.0 ± 0.1	1.3 ± 0.1	<0.001
LDL-C (mM)	3.1 ± 0.9	2.8 ± 0.6	0.008
NAFLD (%)	110(73.3)	33 (21.4)	<0.001
Fasting serum insulin (μIU/mL)	14.3 ± 7.2	9.2 ± 5.0	<0.001
Estradiol (pg/ml)	23.2 ± 7.2	27.0 ± 7.2	<0.001
HOMA-IR	3.5 ± 1.8	2.1 ± 1.3	<0.001

*Abbreviations BMI* body mass index, *FBG* fasting blood glucose, *HbA1C* hemoglobin A1C, *HDL* high density lipoprotein, *LDL* low-density lipoprotein, *NAFLD* nonalcoholic fatty liver disease

Data are mean ± SD. Comparisons were performed by *t*-test

*p* < 0.05 indicates a significant difference from the normal control

### Allele frequencies and genotype distribution

Genotype distribution for each of the four SNPs was compatible with the Hardy–Weinberg equilibrium (*p* > 0.05) in MetS and control patients. The Chi-square test of XbaI genotype’s frequencies in the case and control groups can be used to calculate statistical power. The test power was calculated (power = 1), indicating that the allele frequencies and genotype distributions of MetS patients and controls (Table 2) led to credible conclusions.

**Table 2:** Allele frequencies and genotype distributions of ESR1 and ESR2 gene polymorphisms in the metabolic syndrome (MetS) and control groups

Variables	MetS	Control	P	*OR*(95 % *CI*)^a^
	n (%)	n (%)		
	(*n* = 150)	(*n* = 154)		
ESR1				
XbaI (A > G)				
A allele	131 (43.7)	171 (55.5)		
G allele	169 (56.3)	137 (44.5)	0.004	1.610 (1.169, 2.18)
1 (AA)	29 (19.3)	45 (29.2)		
2 (AG)	73 (48.7)	81 (52.6)	0.894	1.044 (0.556, 1.960)
3 (GG)	48 (32.0)	28 (18.2)	0.109	2.660 (0.874, 3.829)
PVUII (T > C)				
T allele	143 (47.7)	157 (52.3)		
C allele	158 (51.3)	150 (48.7)	0.223	1.219 (0.887, 1.676)
1 (TT)	31 (20.7)	38 (24.7)		
2 (TC)	79 (52.7)	84 (54.5)	0.012	2.440 (1.216, 4.897)
3 (CC)	40 (26.7)	32 (20.8)	0.038	2.288 (1.045, 5.009)
ESR2				
RsaI (B5) (1082A > G)				
A allele	101 (33.7)	99 (32.1)		
G allele	199 (66.3)	209 (67.9)	0.689	1.071 (0.764, 1.503)
1 (AA)	20 (13.3)	18 (11.7)		
2 (AG)	61 (40.7)	63 (40.9)	0.863	1.074 (0.476, 2.426)
3 (GG)	69 (46.0)	73 (47.4)	0.834	0.851 (0.412, 2.044)
AluI (B8) (1730A > G)				
A allele	100 (33.3)	96 (31.2)		
G allele	200 (66.7)	212 (68.8)	0.568	1.104 (0.786, 1.552)
1 (AA)	21 (14.0)	20 (13.0)		
2 (AG)	58 (38.7)	56 (36.4)	0.225	0.592 (0.254, 1.382)
3 (GG)	71 (47.3)	78 (50.6)	0.172	0.567 (0.251, 1.281)

*Abbreviations*: *CI* confidence interval, *OR* odds ratio, *NA* not available

*OR* OR value of rectified BMI and waist circumference for every allele

^a^95% of the 95% CI for the OR CI

*p* < 0.05 indicated statistical significance

G allele frequency in the XbaI polymorphism was markedly elevated in MetS patients compared with control individuals (*p* = 0.004; OR = 1.610, 95%CI 1.169–2.18).

Genotype distributions of the XbaI, PvuII, RsaI and AluI polymorphisms, and allele frequencies of the PvuII, RsaI and AluI polymorphisms were comparable between MetS patients and controls (*p* > 0.05).

### Haplotype distributions of the four SNPs

LD assessment of all SNP pairs revealed XbaI and PvuII of ESR1 were associated,as well as RsaI (B5) and AluI (B8) of ESR2 (Additional file 1: Table S1). Haplotype distributions of the 4 SNPs (PvuII, XbaI, RsaI and AluI) are shown in Table 3. The exclusion criteria was set as frequency < 0.03. ESR1 and ESR2 haplotype frequency distributions were greater than 3%. Analysis showed that XbaI–PvuII based haplotypes were associated with MetS (global *p* = 0.022), while those constructed from RsaI–AluI had no significant associations with MetS (*p* > 0.05). The haplotypes A–T (*p* = 0.015; OR = 0.638, 95%CI 0.443–0.919) and G–C (*p* = 0.024; OR = 1.512, 95%CI 1.055–2.168) obtained from XbaI–PvuII showed significant differences.

**Table 3:** Estimated haplotype distributions in the metabolic syndrome (MetS) and control groups

SNP	Haplotype	MetS (freq.)	Control (freq.)	Global *p*-value	*p*-value
ESR1	A-C	65.15 (0.217)	76.73 (0.249)	0.022	0.351
XbaI-PvuII	A-T	65.85 (0.220)	94.27 (0.306)		0.015
	G-C	93.85 (0.313)	71.27 (0.231)		0.024
	G-T	75.15 (0.250)	65.73 (0.213)		0.279
ESR2	A-A	37.99 (0.127)	25.20 (0.082)	0.271	0.070
1082A > G-1730A > G	A-G	63.01 (0.210)	73.8 (0.240)		0.383
	G-A	62.01 (0.207)	70.8 (0.230)		0.489
	G-G	136.99 (0.457)	138.20 (0.44)		0.845

*Abbreviation*: *SNP* single-nucleotide polymorphism

Haplotypes have frequencies ≥3% in the MetS and control groups

*p* < 0.05 indicated statistical significance

### Associations of genotypes with clinical characteristics

Associations of the XbaI polymorphism with MetS components are shown in Table 4. Patients harboring the G allele in both homozygous and heterozygous forms (i.e. GG or AG) had elevated BMI, waist circumference, systolic and diastolic BPs, FBG, HOMA-IR, TC, TG, LDL-C and NAFLD (%), and lower HDL-C. Among subjects with the PvuII polymorphism, those harboring the minor C allele in both homozygous and heterozygous forms (i.e. CC or TC) showed elevated BMI, fasting serum insulin and HOMA-IR (Additional file 1: Table S5). Associations of XbaI, PvuII and MetS were confirmed by multivariable regression analysis (Table 5). Among subjects with the RsaI polymorphism, those harboring the minor G allele in both homozygous and heterozygous forms showed elevated TG, LDL-C, fasting serum insulin and HOMA-IR (Additional file 1: Table S3). Among subjects with the AluI polymorphism, those carrying the minor G allele in both homozygous and heterozygous forms (AG or GG) showed elevated TC and LDL-C (Additional file 1: Table S4).

**Table 4:** Associations of XbaI genotypes with various clinical characteristics

Variables	AA (*n* = 74)	AG (*n* = 154)	GG (*n* = 76)
Age (years)	62.14 ± 4.24	63.24 ± 4.85	63.11 ± 3.75
BMI (kg/m^2^)	22.97 ± 2.99	23.98 ± 3.40*	24.71 ± 3.18*
Waist circumference (cm)	84.26 ± 5.36	84.92 ± 5.13	86.07 ± 4.96*
FBG (mM)	4.95 ± 0.88	5.21 ± 1.41*	5.70 ± 1.10*#
HbA1C (%)	5.89 ± 1.00	5.94 ± 1.05	6.09 ± 0.98
Systolic blood pressure (mmHg)	132.31 ± 14.39	134.15 ± 13.96	139.05 ± 14.12*#
Diastolic blood pressure (mmHg)	79.95 ± 11.96	81.14 ± 11.39	85.86 ± 10.69*#
Triglycerides (mM)	1.84 ± 0.92	2.08 ± 1.20*	2.38 ± 1.16*
Total cholesterol (mM)	4.29 ± 1.20	4.72 ± 0.83*	5.53 ± 1.12*#
HDL-C (mM)	1.25 ± 0.26	1.15 ± 0.21*	1.06 ± 0.20*#
LDL-C (mM)	2.74 ± 0.64	2.83 ± 0.67	3.39 ± 0.83*#
NAFLD (%)	28 (37.8)	70 (45.5)	45 (59.2)*
Fasting serum insulin (μIU/mL)	11.15 ± 5.68	11.54 ± 6.23	12.54 ± 8.31
Estradiol (pg/ml)	26.82 ± 6.99	24.66 ± 7.48	24.49 ± 7.67
HOMA-IR	2.47 ± 1.32	2.72 ± 1.77*	3.20 ± 1.70*

Measurement data are mean ± SD, and were assessed by one way ANOVA with post-hoc SNK test

*Significant difference from patients harboring AA (*p* < 0.05)

#Significant difference from patients harboring AG (*p* < 0.05). Enumeration count data were described as rate, and assessed by the chi-square test

**Table 5:** Multivariable logistic regression analysis with MetS as the dependent variable

Risk factor	OR (95%CI)	P
BMI	6.541 (2.537,16.864)	< 0.001
Triglycerides	2.386 (1.238,4.599)	0.009
XbaI		
AA (reference)	–	–
AG	1.505 (0.819,2.764)	0.188
GG	2.330 (1.161,4.679)	0.017
PVUII		
TT (reference)	–	–
TC	2.235 (1.120,4.461)	0.023
CC	2.679 (1.225,5.855)	0.014

*Abbreviations*: *BMI* body mass index, *CI* confidence interval, *OR* odds ratio

*p* < 0.05 indicated statistical significance

Regarding XbaI, the control group showed significantly different FBG, TG, TC, HDL-C and LDL-C among the three genotypes (all *p* < 0.05). In MetS patients, BMI, TG, TC and LDL-C were markedly different (Additional file 1: Table S2). As for RsaI (B5), the control group showed significant differences in fasting insulin and HOMA-IR among the three genotypes (all *p* < 0.05); in MetS patients, LDL-C and HOMA-IR were significantly different (Additional file 1: Table S3). As for AluI (B8), BMI and TC significantly differed among the three genotypes in control patients (all *p* < 0.05); in MetS patients, age and TG significantly differed (Additional file 1: Table S4). Regarding PvuII, estradiol levels were significantly different among the three genotypes in control patients (all *p* < 0.05); in MetS patients, age, BMI, systolic BP, HDL-C and HOMA-IR were significantly different (Additional file 1: Table S5).

## Discussion

Changing lifestyles, socioeconomic status, and dietary habits have likely contributed to a rapid increase in the incidence of MetS, which is currently considered a major threat to human health worldwide [21]. High MetS prevalence is found among postmenopausal women [22]. Associations of several polymorphisms in the ESR1 and ESR2 genes with MetS or related components have been described [23]. These associations were also reported in the Study of Women’s Health Across the Nation (SWAN) trial of perimenopausal African-American, Caucasian, Chinese, and Japanese women between 42 and 52 years old [24]. The current study revealed positive associations of ESR1 polymorphisms with MetS in postmenopausal Chinese Han women. Subjects harboring the G allele of XbaI, or the A-T and G-C haplotypes of XbaI–PvuII polymorphisms of ESR1, showed elevated risk of MetS. Similar results were obtained in studies of women of different ages [25, 26]. Two ESR2 subtypes, including RsaI and AluI, were not significantly associated with Mets in this study.

ERb^_^/^_^ mice show normal or improved glucose tolerance and insulin release compared with wild type counterparts [27]. Meanwhile, ERa^_^/^_^ animals display glucose intolerance and insulin resistance [12]. ESR1 and ESR2 are widely distributed in various organs and tissues related to sugar metabolism. Associations of ESR1 polymorphisms and (T2DM) were demonstrated [28]. ESR2 polymorphisms are associated with MetS in Chinese and Japanese populations; in addition, SNPs in the ESR1 show associations with insulin sensitivity in Asian women [24]. ER-α can exert co-modulatory functions with insulin receptor substrate 1 (IRS1) to modulate insulin signaling [29]. Previous studies suggested an inhibitory effect of ERb on glucose transporter four (GLUT4) expression, and revealed that unchecked ERb activity could result in diabetes [25, 30]. As shown above, patients harboring the minor alleles of XbaI and PvuII gene polymorphisms, in both homozygous (GG and CC) or heterozygous (AG and TC) forms, had higher FBG, fasting serum insulin and HOMA-IR. Meanwhile, those carrying the RsaI polymorphism of ESR2 (homozygous or heterozygous) had lower fasting serum insulin and HOMA-IR.

Estrogens have critical functions in lipoprotein metabolism. An imbalance between ESR1 and ESR2 in the adipose tissue could therefore affect the development of metabolic diseases. Epidemiological studies showed hyperlipidemia is a significant risk factor for atherosclerosis, stroke, CAD, and MI [31]. In mice, ERb absence inhibit triglyceride accumulation, maintains normal insulin signaling in the liver and skeletal muscle, and ameliorates whole body insulin sensitivity as well as glucose tolerance [32]. ESR1 polymorphisms are associated with LDL particle size, reduced LDL cholesterol, and TC in adolescent female individuals [33]. SNPs in ESR1 show associations with the degree of adiposity, BMI, and waist circumference [34]. The PvuII T allele of ESR1 and associated genotypes and haplotypes are associated with risk of hyperlipidemia in postmenopausal Chinese Han females [35]. In addition, women with the PvuII T allele show elevated amounts of small LDL particles [36]. Furthermore, postmenopausal women with the T allele have reduced HDL-C and elevated TG, and are more vulnerable to lipid metabolism disorders [37]. These findings were corroborated by several other reports showing associations of ESR1 polymorphisms with BMI; however, associations of ESR2 polymorphisms with hyperlipidemia remain unclear. A study showed that homozygotes for the AluI (rs4986938) of ESR2 have higher BMI, serum TG and apolipoprotein B, and reduced HDL-C [38]. In mice, ESR2 deficiency is associated with abnormal vascular function and hypertension [39]. ESR2 polymorphisms are also associated with hypertension in postmenopausal Japanese women [40]. As shown above, positive associations were obtained of the G allele of XbaI polymorphism of the ESR1 gene with elevated TG, TC, LDL-C levels, as well as lower HDL-C, in postmenopausal Chinese women. The RsaI and AluI polymorphisms of ESR2 exhibited associations with TG, TC and LDL-C amounts.

## Conclusion

Currently, MetS incidence is higher in postmenopausal women compared with age—matched males. Interventions to halt the rapidly increasing MetS incidence are of great clinical interest. Several studies have assessed associations of ESR1 and ESR2 with MetS or related components in different populations, but have often yielded conflicting findings. Furthermore, very few such studies have evaluated Chinese subjects. We here demonstrated associations of ESR1 and ESR2 polymorphisms with MetS and related components, especially obesity, and lipid and glucose metabolism, in postmenopausal Chinese Han females. Meanwhile, the G allele frequency of the XbaI polymorphism was markedly elevated in MetS patients compared with the control group. The RsaI and AluI polymorphisms of the ESR2 gene were not associated with MetS. In addition, XbaI and PvuII polymorphisms of ESR1 were associated with some MetS components. The current findings could include biases related to sample size, age, and ethnicity. Nevertheless, the present data could provide valuable insights into MetS epidemiology, particularly in Han Chinese individuals, providing a basis for the development of novel therapeutic interventions.

## Additional files

Additional file 1: **Table S1.** Linkage disequilibrium tests (D’ and r^2^). **Table S2.** Relationship between Xba1 genotypes and clinical indicators within each group. **Table S3.** Relationship between RsaI (B5) genotypes and clinical indicators in each group. **Table S4.** Relationship between AluI (B8) genotypes and clinical indicators of each group. **Table S5.** Relationship between PVUII genotypes and clinical indicators in each group. **Figure S1**. Genotype validation by direct sequencing for PvuII and XbaI. **Figure S2.** Genotype validation by direct sequencing for RsaI. **Figure S3**. Genotype validation by direct sequencing for AluI. (DOCX 430 kb)

## Abbreviations

ATP III: Adult Treatment Panel III
BMI: Body mass index
BP: Blood pressure
CVD: Cardiovascular disease
DM: Diabetes mellitus
E2: Estradiol
ER: Estrogen receptor
ESR: Estrogen receptor
FBG: Fasting blood glucose
GLUT4: Glucose transporter four
HbA1C: Hemoglobin A1C
HDL-C: High-density lipoprotein cholesterol
HOMA-IR: Homeostasis model assessment of insulin resistance
IRS1: Insulin receptor substrate 1
LDL-C: Low density lipoprotein
MetS: Metabolic syndrome
MI: Myocardial infarction
NAFLD: Non-alcoholic fatty liver disease
SNPs: Single-nucleotide polymorphisms
SWAN: Study of Women’s Health Across the Nation
T2DM: Type 2 diabetes mellitus
TC: Total cholesterol
TG: Triglyceride

## References

[1] Fahed AC, El-Hage-Sleiman AK, Farhat TI (2012). Diet, genetics, and disease: a focus on the Middle East and North Africa region. J Nutr Metab.

[2] Li R, Li W, Lun Z (2016). Prevalence of metabolic syndrome in mainland China: a meta-analysis of published studies. BMC Public Health.

[3] Navaneethan SD, Schold JD, Kirwan JP (2013). Metabolic syndrome, ESRD, and death in CKD. Clin J Am Soc Nephrol.

[4] Wang Y, Jiang T, Wang X (2017). Association between insomnia and metabolic syndrome in a Chinese Han population: a cross-sectional study. Sci Rep.

[5] Grundy SM, Cleeman JI, Daniels SR (2005). Diagnosis and management of the metabolic syndrome: an American Heart Association/National Heart, Lung, and Blood Institute Scientific Statement. Circulation.

[6] Westerman S., Wenger N. K. (2016). Women and heart disease, the underrecognized burden: sex differences, biases, and unmet clinical and research challenges. Clinical Science.

[7] Hidalgo LA, Chedraui PA, Morocho N (2006). The metabolic syndrome among postmenopausal women in Ecuador. Gynecol Endocrinol.

[8] Salpeter SR, Walsh JM, Ormiston TM (2006). Meta-analysis: effect of hormone-replacement therapy on components of the metabolic syndrome in postmenopausal women. Diabetes Obes Metab.

[9] Shakir YA, Samsioe G, Nyberg P (2007). Do sex hormones influence features of the metabolic syndrome in middle-aged women? A population-based study of Swedish women: the Women's health in the Lund area (WHILA) study. Fertil Steril.

[10] Arnal JF, Fontaine C, Abot A (2013). Lessons from the dissection of the activation functions (AF-1 and AF-2) of the estrogen receptor alpha in vivo. Steroids.

[11] Mendelsohn ME, Karas RH (1999). The protective effects of estrogen on the cardiovascular system. N Engl J Med.

[12] Heine PA, Taylor JA, Iwamoto GA (2000). Increased adipose tissue in male and female estrogen receptor-alpha knockout mice. Proc Natl Acad Sci U S A.

[13] Takeda K, Toda K, Saibara T (2003). Progressive development of insulin resistance phenotype in male mice with complete aromatase (CYP19) deficiency. J Endocrinol.

[14] Barros RPA, Gustafsson J-Å (2011). Estrogen receptors and the metabolic network. Cell Metab.

[15] Zheng Yonglan, Huo Dezheng, Zhang Jing, Yoshimatsu Toshio F., Niu Qun, Olopade Olufunmilayo I. (2012). Microsatellites in the Estrogen Receptor (ESR1, ESR2) and Androgen Receptor (AR) Genes and Breast Cancer Risk in African American and Nigerian Women. PLoS ONE.

[16] Hayes DF, Skaar TC, Rae JM (2010). Estrogen receptor genotypes, menopausal status, and the effects of tamoxifen on lipid levels: revised and updated results. Clin Pharmacol Ther.

[17] Nilsson M, Naessén S, Dahlman I (2004). Association of estrogen receptor beta gene polymorphisms with bulimic disease in women. Mol Psychiatry.

[18] Goulart AC, Zee RY, Pradhan A (2009). Associations of the estrogen receptors 1 and 2 gene polymorphisms with the metabolic syndrome in women. Metab Syndr Relat Disord.

[19] Chinese Diabetes Society (2014). China guideline for type 2 diabetes (2013). Chin J Endocrinol Metab.

[20] Farrell GC, Chitturi S, Lau GK (2007). Guidelines for the assessment and management of non-alcoholic fatty liver disease in the Asia-Pacific region: executive summary. J Gastroenterol Hepatol.

[21] Zimmet P, Magliano D, Matsuzawa Y (2005). The metabolic syndrome: a global public health problem and a new definition. J Atheroscler Thromb.

[22] Ponholzer A, Temml C, Rauchenwald M (2008). Is the metabolic syndrome a risk factor for female sexual dysfunction in sexually active women?. Int J Impot Res.

[23] Lo JC, Zhao X, Scuteri A (2006). The association of genetic polymorphisms in sex hormone biosynthesis and action with insulin sensitivity and diabetes mellitus in women at midlife. Am J Med.

[24] Sowers MR, Wilson AL, Karvonen-Gutierrez CA (2006). Sex steroid hormone pathway genes and health-related measures in women of 4 races/ethnicities: the study of Women's health across the nation (SWAN). Am J Med.

[25] Ramos RG, Olden K (2008). The prevalence of metabolic syndrome among US women of childbearing age. Am J Public Health.

[26] Ghattas MH, Mehanna ET, Mesbah NM (2013). Association of estrogen receptor alpha gene polymorphisms with metabolic syndrome in Egyptian women. Metabolism.

[27] Bryzgalova G, Lundholm L, Portwood N (2008). Mechanisms of antidiabetogenic and body weight-lowering effects of estrogen in high-fat diet-fed mice. Am J Physiol Endocrinol Metab.

[28] Huang Q, Wang TH, Lu WS (2006). Estrogen receptor alpha gene polymorphism associated with type 2 diabetes mellitus and the serum lipid concentration in Chinese women in Guangzhou. Chin Med J.

[29] Andò S, Panno ML, Salerno M (1998). Role of IRS-1 signaling in insulin-induced modulation of estrogen receptors in breast cancer cells. Biochem Biophys Res Commun.

[30] Barros RP, Machado UF, Warner M (2006). Muscle GLUT4 regulation by estrogen receptors ERbeta and ERalpha. Proc Natl Acad Sci U S A.

[31] National Cholesterol Education Program (NCEP) Expert Panel on Detection, Evaluation, and Treatment of High Blood Cholesterol in Adults (Adult Treatment Panel III) (2002). Third report of the National Cholesterol Education Program (NCEP) expert panel on detection, evaluation, and treatment of high blood cholesterol in adults (adult treatment panel III) final report. Circulation.

[32] Foryst-Ludwig A, Clemenz M, Hohmann S (2008). Metabolic actions of estrogen receptor beta (ERbeta) are mediated by a negative cross-talk with PPARgamma. PLoS Genet.

[33] Nordström P, Glader CA, Dahlén G (2003). Oestrogen receptor alpha gene polymorphism is related to aortic valve sclerosis in postmenopausal women. J Intern Med.

[34] Gallagher CJ, Langefeld CD, Gordon CJ (2007). Association of the estrogen receptor-alpha gene with the metabolic syndrome and its component traits in African-American families: the insulin resistance atherosclerosis family study. Diabetes.

[35] Zhao T, Zhang D, Liu Y (2010). Association between ESR1 and ESR2 gene polymorphisms and hyperlipidemia in Chinese Han postmenopausal women. J Hum Genet.

[36] Demissie S, Cupples LA, Shearman AM (2006). Estrogen receptor-alpha variants are associated with lipoprotein size distribution and particle levels in women: the Framingham heart study. Atherosclerosis.

[37] Lamon-Fava S, Asztalos BF, Howard TD (2010). Association of polymorphisms in genes involved in lipoprotein metabolism with plasma concentrations of remnant lipoproteins and HDL subpopulations before and after hormone therapy in postmenopausal women. Clin Endocrinol.

[38] Mansur Ade P, Nogueira CC, Strunz CM (2005). Genetic polymorphisms of estrogen receptors in patients with premature coronary artery disease. Arch Med Res.

[39] Zhu Y, Bian Z, Lu P (2002). Abnormal vascular function and hypertension in mice deficient in estrogen receptor beta. Science.

[40] Ogawa S, Emi M, Shiraki M (2000). Association of estrogen receptor beta (ESR2) gene polymorphism with blood pressure. J Hum Genet.

