# Supplementary material for: Estrogen receptor 1 gene polymorphisms are associated with metabolic syndrome in postmenopausal women in China
Source: BMC Endocr Disord. 2018 Sep 14;18:65. doi: 10.1186/s12902-018-0289-4 (PMC6137943; doi:10.1186/s12902-018-0289-4)

**Supplementary Table S1.** Linkage disequilibrium tests (D’ and r^2^)

| D’ | B5 | B8 | PuvII |
| --- | --- | --- | --- |
| Xba1 | 0.062 | 0.267 | 0.074 |
| B5 | - | 0.027 | 0.263 |
| B8 | - | - | 0.040 |
| r^2^ | B5 | B8 | PuvII |
| Xba1 | 0.002 | 0.034 | 0.005 |
| B5 | - | ＜0.001 | 0.035 |
| B8 | - | - | 0.001 |

The estimation of LD for all pairs SNP makers showed that XbaI and PvuII of ESRl were in a relatively relationship with the standardized D’=0.074，as well as RsaI (B5) and AluI (B8) of ESR2 with the standardized D’=0.027.

**Supplementary Table S2.** Relationship between Xba1 genotypes and clinical indicators within each group

|  | AA | AG | GG |
| --- | --- | --- | --- |
| Total | n=74 | n=154 | n=76 |
| Age (years) | 62.14±4.24 | 63.24±4.85 | 63.11±3.75 |
| **BMI (kg/m2)** | **22.97±2.99** | **23.98±3.40** | **24.71±3.18*** |
| Waist circumference (cm) | 84.26±5.36 | 84.92±5.13 | 86.07±4.96 |
| **FBG (mmol/L)** | **5.82±1.42** | **6.08±1.51** | **6.43±1.33*** |
| HbA1C (%) | 5.89±1.00 | 5.94±1.05 | 6.09±0.98 |
| **SBP (mmHg)** | **132.31±14.39** | **134.15±13.96** | **139.05±14.12*#** |
| **DBP (mmHg)** | **79.95±11.96** | **81.14±11.39** | **85.86±10.69*#** |
| **TG (mmol/L)** | **1.84±0.92** | **2.08±1.20** | **2.38±1.16*** |
| **TC (mmol/L)** | **4.29±1.20** | **4.72±0.83*** | **5.53±1.12*#** |
| **HDL-C (mmol/L)** | **1.25±0.26** | **1.15±0.21*** | **1.06±0.20*#** |
| **LDL-C (mmol/L)** | **2.74±0.64** | **2.83±0.67** | **3.39±0.83*#** |
| NAFLD (%) | 28 (37.8) | 70 (45.5) | 45 (59.2) |
| Fasting serum insulin (μIU/mL) | 11.15±5.68 | 11.54±6.23 | 12.54±8.31 |
| Estradiol (pg/ml) | 26.82±6.99 | 24.66±7.67 | 24.49±7.67 |
| **HOMA-IR** | **2.47±1.32** | **2.72±1.77** | **3.20±1.70*** |
| MetS | n=29 | n=73 | n=48 |
| Age (years) | 61.93±3.70 | 63.55±4.37 | 62.88±3.81 |
| **BMI (kg/m2)** | **24.15±3.53** | **25.46±2.73** | **25.98±2.68*** |
| Waist circumference (cm) | 87.31±7.18 | 88.59±4.69 | 88.33±4.68 |
| FBG (mmol/L) | 7.03±1.39 | 7.14±1.41 | 6.96±1.39 |
| HbA1C (%) | 6.91±0.76 | 6.80±0.87 | 6.52±0.95 |
| SBP (mmHg) | 148.52±6.98 | 147.34±7.21 | 148.88±6.41 |
| DBP (mmHg) | 93.34±4.50 | 91.77±4.65 | 93.04±4.23 |
| **TG (mmol/L)** | **1.94±0.72** | **2.35±1.02** | **2.67±1.18*** |
| **TC (mmol/L)** | **4.77±1.26** | **4.89±0.77** | **5.48±1.09*#** |
| HDL-C (mmol/L) | 0.96±0.08 | 0.97±0.14 | 0.96±0.17 |
| **LDL-C (mmol/L)** | **3.03±0.80** | **2.84±0.78** | **3.41±0.88#** |
| NAFLD (%) | 28 (37.8) | 70 (45.5) | 45 (59.2) |
| Fasting serum insulin (μIU/mL) | 14.28±6.60 | 14.04±6.21 | 14.63±8.89 |
| Estradiol (pg/ml) | 23.31±5.51 | 23.12±7.63 | 23.25±7.68 |
| HOMA-IR | 3.18±1.50 | 3.45±1.90 | 3.63±1.76 |
| Control | n=45 | n=81 | n=28 |
| Age (years) | 62.27±4.58 | 62.96±5.26 | 63.50±3.68 |
| BMI (kg/m^2^) | 22.20±2.31 | 22.65±3.40 | 22.55±2.82 |
| Waist circumference (cm) | 82.29±2.17 | 81.62±2.68 | 82.18±2.36 |
| **FBG (mmol/L)** | **5.04±0.73** | **5.13±0.78** | **5.50±0.40*** |
| HbA1C (%) | 5.22±0.40 | 5.17±0.38 | 5.35±0.44 |
| SBP (mmHg) | 121.87±5.29 | 122.26±4.91 | 122.21±4.10 |
| DBP (mmHg) | 71.31±5.48 | 71.57±5.72 | 73.54±6.10 |
| TG (mmol/L) | 1.78±1.03 | 1.83±1.29 | 1.88±0.96 |
| **TC (mmol/L)** | **3.99±1.07** | **4.56±0.85*** | **5.61±1.19*#** |
| **HDL-C (mmol/L)** | **1.44±0.12** | **1.31±0.10*** | **1.23±0.13*#** |
| **LDL-C (mmol/L)** | **2.54±0.43** | **2.82±0.55*** | **3.35±0.75*#** |
| NAFLD (%) | 28 (37.8) | 70 (45.5) | 45 (59.2) |
| Fasting serum insulin (μIU/mL) | 9.14±3.90 | 9.28±5.36 | 8.95±5.76 |
| Estradiol (pg/ml) | 29.09±6.95 | 26.05±7.11 | 26.61±7.31 |
| HOMA-IR | 2.02±0.97 | 2.07±1.36 | 2.48±1.48 |

Continuous data were presented as mean±SD and analyzed by one-way ANOVA and the Bonferroni method for multiple comparisons between groups.

Categorical data are presented as n (%) and analyzed using chi-square test.

*P<0.05 vs. the AA group

#P<0.05 vs. the AG group

Abbreviations: FBG, fasting blood glucose; HbA1C, hemoglobin A1C; SBP, systolic blood pressure; DBP, diastolic blood pressure; TG, Triglycerides; TC, total cholesterol; HDL-C, high-density lipoprotein cholesterol; LDL-C, low-density lipoprotein cholesterol; NAFLD, non-alcoholic fatty liver disease; HOMA-IR, homeostasis model assessment of insulin resistance.

**Supplementary Table S3.** Relationship between RsaI (B5) genotypes and clinical indicators in each group

|  | AA | AG | GG |
| --- | --- | --- | --- |
| Total | n=38 | n=124 | n=142 |
| **Age (years)** | **62.00±4.63** | **63.71±4.42*** | **62.51±4.38** |
| BMI (kg/m2) | 24.05±4.67 | 23.72±3.08 | 24.05±3.04 |
| Waist circumference (cm) | 85.90±4.62 | 84.33±5.19 | 85.44±5.25 |
| FBG (mmol/L) | 6.30±1.72 | 6.10±1.47 | 6.05±1.37 |
| HbA1C (%) | 5.80±0.84 | 5.97±0.99 | 6.01±1.09 |
| SBP (mmHg) | 137.11±14.45 | 134.81±13.80 | 134.45±14.70 |
| DBP (mmHg) | 82.29±12.73 | 82.29±11.82 | 81.73±11.05 |
| **TG (mmol/L)** | **2.52±1.35** | **2.01±1.09*** | **2.09±1.01** |
| TC (mmol/L) | 4.98±1.01 | 4.79±1.15 | 4.80±1.08 |
| HDL-C (mmol/L) | 1.11±0.23 | 1.16±0.24 | 1.16±0.23 |
| **LDL-C (mmol/L)** | **2.55±0.73** | **3.06±0.80*** | **2.96±0.68*** |
| NAFLD (%) | 28 (37.8) | 70 (45.5) | 45 (59.2) |
| **Fasting serum insulin (μIU/mL)** | **14.86±8.13** | **10.53±5.99*** | **11.86±6.60*** |
| Estradiol (pg/ml) | 24.79±9.15 | 25.69±7.23 | 24.76±7.16 |
| **HOMA-IR** | **3.83±2.41** | **2.48±1.51*** | **2.77±1.45*** |
| MetS | n=20 | n=61 | n=69 |
| Age (years) | 62.10±3.13 | 63.49±4.25 | 62.87±4.19 |
| BMI (kg/m2) | 25.54±2.81 | 25.57±2.70 | 25.14±3.19 |
| Waist circumference (cm) | 88.80±4.06 | 87.10±5.88 | 89.13±4.79 |
| FBG (mmol/L) | 7.42±1.68 | 7.06±1.38 | 6.96±1.32 |
| HbA1C (%) | 6.35±0.80 | 6.71±0.85 | 6.86±0.92 |
| SBP (mmHg) | 149.45±7.78 | 147.36±7.32 | 148.28±6.28 |
| DBP (mmHg) | 93.40±4.36 | 93.03±4.44 | 91.72±4.58 |
| TG (mmol/L) | 2.63±0.76 | 2.36±1.27 | 2.31±0.90 |
| TC (mmol/L) | 5.01±0.72 | 5.08±1.02 | 5.05±1.11 |
| HDL-C (mmol/L) | 0.93±1.12 | 0.97±0.15 | 0.97±0.14 |
| **LDL-C (mmol/L)** | **2.40±0.71** | **3.28±0.89*** | **3.07±0.76*** |
| NAFLD (%) | 28 (37.8) | 70 (45.5) | 45 (59.2) |
| Fasting serum insulin (μIU/mL) | 17.90±8.96 | 13.89±6.27 | 13.57±7.20 |
| Estradiol (pg/ml) | 25.00±10.91 | 23.82±6.81 | 22.13±6.18 |
| **HOMA-IR** | **4.91±2.71** | **3.28±1.54*** | **3.18±1.39*** |
| Control | n=18 | n=63 | n=73 |
| Age (years) | 61.89±5.98 | 63.92±4.60 | 62.18±4.55 |
| BMI (kg/m2) | 22.39±5.76 | 21.93±2.27 | 23.02±2.52 |
| Waist circumference (cm) | 82.67±2.66 | 81.65±2.25 | 81.96±2.63 |
| FBG (mmol/L) | 5.06±0.43 | 5.17±0.80 | 5.20±0.72 |
| HbA1C (%) | 5.18±0.26 | 5.25±0.41 | 5.20±0.42 |
| SBP (mmHg) | 123.39±2.83 | 122.65±4.52 | 121.38±5.44 |
| DBP (mmHg) | 69.94±5.00 | 71.89±5.89 | 72.29±5.76 |
| TG (mmol/L) | 2.40±1.81 | 1.68±1.01 | 1.81±1.05 |
| TC (mmol/L) | 4.96±1.29 | 4.50±1.20 | 4.56±1.00 |
| HDL-C (mmol/L) | 1.30±0.14 | 1.34±0.15 | 1.34±0.12 |
| LDL-C (mmol/L) | 2.71±0.74 | 2.85±0.64 | 2.86±0.58 |
| NAFLD (%) | 28 (37.8) | 70 (45.5) | 45 (59.2) |
| **Fasting serum insulin (μIU/mL)** | **11.48±5.59** | **7.29±3.37*** | **10.25±5.57#** |
| Estradiol (pg/ml) | 24.56±7.01 | 27.51±7.20 | 27.25±7.17 |
| **HOMA-IR** | **2.64±1.24** | **1.69±0.99*** | **2.38±1.42#** |

Continuous data were presented as mean±SD and analyzed by one-way ANOVA and the Bonferroni method for multiple comparisons between groups.

Categorical data are presented as n (%) and analyzed using chi-square test.

*P<0.05 vs. the AA group

#P<0.05 vs. the AG group

Abbreviations: FBG, fasting blood glucose; HbA1C, hemoglobin A1C; SBP, systolic blood pressure; DBP, diastolic blood pressure; TG, Triglycerides; TC, total cholesterol; HDL-C, high-density lipoprotein cholesterol; LDL-C, low-density lipoprotein cholesterol; NAFLD, non-alcoholic fatty liver disease; HOMA-IR, homeostasis model assessment of insulin resistance.

**Supplementary Table S4.** Relationship between AluI (B8) genotypes and clinical indicators of each group

|  | AA | AG | GG |
| --- | --- | --- | --- |
| Total | n=41 | n=114 | n=149 |
| Age (years) | 61.66±3.40 | 63.01±5.04 | 63.23±4.20 |
| BMI (kg/m2) | 23.12±3.74 | 24.28±3.15 | 23.86±3.25 |
| Waist circumference (cm) | 85.22±5.07 | 84.93±5.51 | 85.09±4.96 |
| FBG (mmol/L) | 5.88±1.42 | 6.09±1.46 | 6.18±1.46 |
| HbA1C (%) | 6.07±0.95 | 6.02±1.08 | 5.90±0.99 |
| SBP (mmHg) | 135.10±14.64 | 135.40±15.30 | 134.52±13.43 |
| DBP (mmHg) | 82.61±12.11 | 82.35±11.57 | 81.62±11.44 |
| TG (mmol/L) | 1.70±0.73 | 2.20±1.13 | 2.13±1.22 |
| **TC (mmol/L)** | **4.22±1.24** | **4.89±1.07*** | **4.92±1.03*** |
| HDL-C (mmol/L) | 1.16±0.22 | 1.13±0.24 | 1.17±0.23 |
| **LDL-C (mmol/L)** | **2.68±0.64** | **2.94±0.80** | **3.03±0.73*** |
| NAFLD (%) | 28 (37.8) | 70 (45.5) | 45 (59.2) |
| Fasting serum insulin (μIU/mL) | 11.83±5.46 | 11.56±6.52 | 11.75±7.14 |
| Estradiol (pg/ml) | 26.71±6.21 | 25.19±7.72 | 24.68±7.53 |
| HOMA-IR | 2.74±1.37 | 2.60±1.57 | 2.94±1.81 |
| MetS | n=21 | n=58 | n=71 |
| **Age (years)** | **60.95±3.14** | **63.59±4.30*** | **63.17±4.03** |
| BMI (kg/m2) | 25.14±3.90 | 25.91±2.90 | 25.00±2.60 |
| Waist circumference (cm) | 87.90±5.40 | 87.88±6.01 | 88.68±4.51 |
| FBG (mmol/L) | 6.70±1.35 | 7.08±1.30 | 7.15±1.48 |
| HbA1C (%) | 6.76±0.77 | 6.81±0.93 | 6.66±0.88 |
| SBP (mmHg) | 147.33±8.01 | 149.28±6.73 | 147.28±6.66 |
| DBP (mmHg) | 93.24±4.30 | 92.47±4.70 | 92.27±4.47 |
| **TG (mmol/L)** | **1.57±0.54** | **2.39±1.09*** | **2.60±1.02*** |
| TC (mmol/L) | 4.67±1.16 | 5.07±0.97 | 5.16±1.02 |
| HDL-C (mmol/L) | 0.97±0.07 | 0.94±0.15 | 0.98±0.15 |
| LDL-C (mmol/L) | 2.70±0.66 | 3.05±0.89 | 3.18±0.85 |
| NAFLD (%) | 28 (37.8) | 70 (45.5) | 45 (59.2) |
| Fasting serum insulin (μIU/mL) | 13.65±4.29 | 14.76±6.77 | 14.06±8.21 |
| Estradiol (pg/ml) | 26.33±6.04 | 23.35±6.63 | 22.16±7.83 |
| HOMA-IR | 3.15±1.03 | 3.36±1.67 | 3.62±1.99 |
| Control | n=20 | n=56 | n=78 |
| Age (years) | 62.40±3.59 | 62.41±5.69 | 63.29±4.38 |
| **BMI (kg/m2)** | **20.99±2.06** | **22.58±2.44** | **22.83±3.45*** |
| Waist circumference (cm) | 82.40±2.64 | 81.88±2.49 | 81.82±2.46 |
| FBG (mmol/L) | 5.02±0.89 | 5.05±0.73 | 5.30±0.66 |
| HbA1C (%) | 5.35±0.45 | 5.20±0.39 | 5.20±0.39 |
| SBP (mmHg) | 122.25±6.66 | 121.02±4.54 | 122.91±4.45 |
| DBP (mmHg) | 71.45±5.77 | 71.88±5.72 | 71.94±5.81 |
| TG (mmol/L) | 1.84±0.88 | 2.00±1.14 | 1.70±1.23 |
| **TC (mmol/L)** | **3.74±1.16** | **4.71±1.14*** | **4.71±1.01*** |
| HDL-C (mmol/L) | 1.36±0.11 | 1.32±0.15 | 1.34±0.13 |
| LDL-C (mmol/L) | 2.66±0.63 | 2.82±0.68 | 2.90±0.57 |
| NAFLD (%) | 28 (37.8) | 70 (45.5) | 45 (59.2) |
| Fasting serum insulin (μIU/mL) | 9.93±5.99 | 8.25±4.24 | 9.66±5.25 |
| Estradiol (pg/ml) | 27.10±6.51 | 27.11±8.34 | 26.97±6.50 |
| HOMA-IR | 2.31±1.57 | 1.80±0.96 | 2.32±1.37 |

Continuous data were presented as mean±SD and analyzed by one-way ANOVA and the Bonferroni method for multiple comparisons between groups.

Categorical data are presented as n (%) and analyzed using chi-square test.

*P<0.05 vs. the AA group

#P<0.05 vs. the AG group

Abbreviations: FBG, fasting blood glucose; HbA1C, hemoglobin A1C; SBP, systolic blood pressure; DBP, diastolic blood pressure; TG, Triglycerides; TC, total cholesterol; HDL-C, high-density lipoprotein cholesterol; LDL-C, low-density lipoprotein cholesterol; NAFLD, non-alcoholic fatty liver disease; HOMA-IR, homeostasis model assessment of insulin resistance.

**Supplementary Table S5.** Relationship between PVUII genotypes and clinical indicators in each group

|  | TT | TC | CC |
| --- | --- | --- | --- |
| Total | n=69 | n=163 | n=72 |
| Age (years) | 63.14±5.92 | 63.07±4.02 | 62.43±3.76 |
| **BMI (kg/m2)** | **25.17±3.69** | **23.21±3.15*** | **24.32±2.79#** |
| Waist circumference (cm) | 84.93±4.72 | 85.07±5.69 | 85.10±4.34 |
| FBG (mmol/L) | 5.86±1.35 | 6.11±1.43 | 6.32±1.57 |
| HbA1C (%) | 5.96±1.08 | 5.94±1.01 | 6.02±0.98 |
| SBP (mmHg) | 133.12±12.02 | 134.80±14.80 | 136.96±14.99 |
| DBP (mmHg) | 80.46±11.12 | 81.92±11.81 | 83.78±11.29 |
| TG (mmol/L) | 2.03±1.05 | 2.11±1.19 | 2.11±1.11 |
| TC (mmol/L) | 4.76±1.13 | 4.84±1.08 | 4.81±1.12 |
| HDL-C (mmol/L) | 1.12±0.24 | 1.18±0.22 | 1.12±0.24 |
| LDL-C (mmol/L) | 2.93±0.62 | 2.95±0.76 | 2.97±0.84 |
| NAFLD (%) | 28 (37.8) | 70 (45.5) | 45 (59.2) |
| **Fasting serum insulin (μIU/mL)** | **10.29±4.72** | **11.59±6.45** | **13.27±8.37*** |
| Estradiol (pg/ml) | 24.54±7.67 | 25.61±7.39 | 24.68±7.41 |
| **HOMA-IR** | **2.21±1.13** | **2.71±1.44** | **3.48±2.26*#** |
| MetS | n=31 | n=79 | n=40 |
| **Age (years)** | **64.35±5.16** | **63.09±3.72** | **61.85±3.59*#** |
| **BMI (kg/m2)** | **27.85±3.02** | **24.19±2.83*** | **25.78±1.42*#** |
| Waist circumference (cm) | 87.87±5.08 | 88.87±5.86 | 87.35±3.83 |
| FBG (mmol/L) | 6.92±1.09 | 7.02±1.46 | 7.25±1.48 |
| HbA1C (%) | 6.90±0.92 | 6.75±0.84 | 6.57±0.94 |
| **SBP (mmHg)** | **144.81±7.34** | **148.73±6.67*** | **149.25±6.42*** |
| DBP (mmHg) | 91.55±4.98 | 92.72±4.46 | 92.73±4.29 |
| TG (mmol/L) | 2.23±0.80 | 2.35±1.11 | 2.53±1.11 |
| TC (mmol/L) | 4.97±0.90 | 5.09±1.05 | 5.05±1.07 |
| **HDL-C (mmol/L)** | **0.90±0.12** | **1.01±0.14*** | **0.95±0.13** |
| LDL-C (mmol/L) | 3.08±0.62 | 3.03±0.87 | 3.11±0.97 |
| NAFLD (%) | 28 (37.8) | 70 (45.5) | 45 (59.2) |
| Fasting serum insulin (μIU/mL) | 12.62±4.74 | 14.04±7.10 | 16.01±8.63 |
| Estradiol (pg/ml) | 24.87±9.28 | 22.75±6.37 | 22.80±7.07 |
| **HOMA-IR** | **2.63±1.03** | **3.30±1.50** | **4.39±2.25*#** |
| Control | n=38 | n=84 | n=32 |
| Age (years) | 62.16±6.37 | 63.06±4.30 | 63.16±3.90 |
| BMI (kg/m2) | 22.99±2.61 | 22.28±3.17 | 22.49±3.01 |
| Waist circumference (cm) | 82.53±2.61 | 81.50±2.07 | 82.28±3.14 |
| FBG (mmol/L) | 4.98±0.81 | 5.26±0.70 | 5.16±0.66 |
| HbA1C (%) | 5.20±0.37 | 5.18±0.37 | 5.34±0.50 |
| SBP (mmHg) | 123.58±3.71 | 121.69±5.22 | 121.59±4.89 |
| DBP (mmHg) | 71.42±4.48 | 71.76±6.17 | 72.59±5.98 |
| TG (mmol/L) | 1.87±1.20 | 1.90±1.23 | 1.59±0.88 |
| TC (mmol/L) | 4.59±1.27 | 4.61±1.06 | 4.51±1.12 |
| HDL-C (mmol/L) | 1.31±0.12 | 1.34±0.14 | 1.34±0.15 |
| LDL-C (mmol/L) | 2.81±0.61 | 2.87±0.64 | 2.79±0.61 |
| NAFLD (%) | 28 (37.8) | 70 (45.5) | 45 (59.2) |
| Fasting serum insulin (μIU/mL) | 8.40±3.81 | 9.29±4.79 | 9.84±6.70 |
| **Estradiol (pg/ml)** | **24.26±6.16** | **28.30±7.30*** | **27.03±7.36** |
| HOMA-IR | 1.88±1.11 | 2.16±1.15 | 2.34±1.73 |

Continuous data were presented as mean±SD and analyzed by one-way ANOVA and the Bonferroni method for multiple comparisons between groups.

Categorical data are presented as n (%) and analyzed using chi-square test.

*P<0.05 vs. the AA group

#P<0.05 vs. the AG group

Abbreviations: FBG, fasting blood glucose; HbA1C, hemoglobin A1C; SBP, systolic blood pressure; DBP, diastolic blood pressure; TG, Triglycerides; TC, total cholesterol; HDL-C, high-density lipoprotein cholesterol; LDL-C, low-density lipoprotein cholesterol; NAFLD, non-alcoholic fatty liver disease; HOMA-IR, homeostasis model assessment of insulin resistance.

**Supplementary Figure S1.** Genotype validation by direct sequencing for PvuII and XbaI.


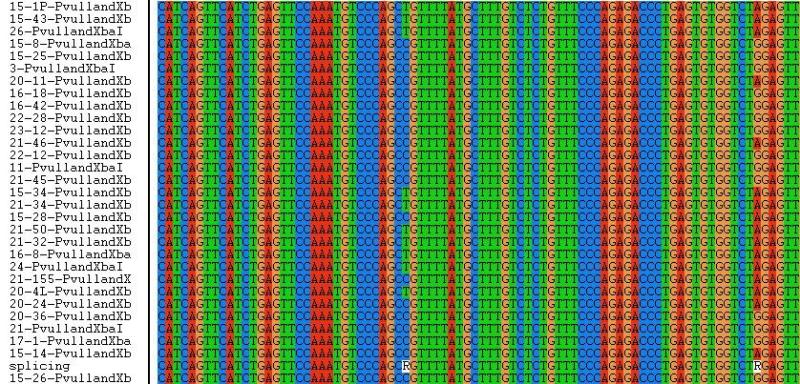


**Supplementary Figure S2.** Genotype validation by direct sequencing for RsaI.


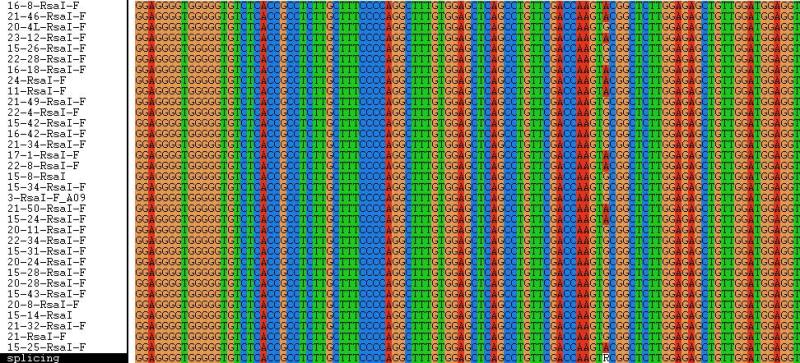


**Supplementary Figure S3.** Genotype validation by direct sequencing for AluI.


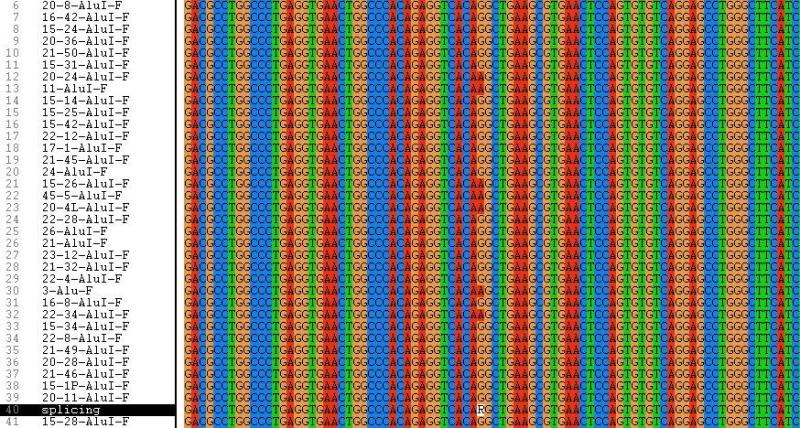

Supplement: Supplementary file 1 — Table S1. Linkage disequilibrium tests (D’ and r2). Table S2. Relationship between Xba1 genotypes and clinical indicators within each group. Table S3. Relationship between RsaI (B5) genotypes and clinical indicators in each group. Table S4. Relationship between AluI (B8) genotypes and clinical indicators of each group. Table S5. Relationship between PVUII genotypes and clinical indicators in each group. Figure S1. Genotype validation by direct sequencing for PvuII and XbaI. Figure S2. Genotype validation by direct sequencing for RsaI. Figure S3. Genotype validation by direct sequencing for AluI. (DOCX 430 kb) [file 12902_2018_289_MOESM1_ESM.docx]
